# Biomarkers of gut injury in neonates – where are we in predicting necrotising enterocolitis?

**DOI:** 10.3389/fped.2022.1048322

**Published:** 2022-11-28

**Authors:** Claire Howarth, Jayanta Banerjee, Simon Eaton, Narendra Aladangady

**Affiliations:** ^1^ Neonatal Unit, Homerton Healthcare NHS Foundation Trust, London, United Kingdom; ^2^ Neonatal Unit, Imperial College Healthcare NHS Trust and Imperial College London, London, United Kingdom; ^3^ University College London Great Ormond Street Institute of Child Health, London, England; ^4^ Barts and The London School of Medicine and Dentistry, Queen Mary University of London (QMUL), London, United Kingdom

**Keywords:** NEC, biomarker, gut oxygenation, tissue injury, ischaemia

## Abstract

Despite advances in neonatal care Necrotising Enterocolitis (NEC) continues to have a significant mortality and morbidity rate, and with increasing survival of those more immature infants the population at risk of NEC is increasing. Ischaemia, reperfusion, and inflammation underpin diseases affecting intestinal blood flow causing gut injury including Necrotising Enterocolitis. There is increasing interest in tissue biomarkers of gut injury in neonates, particularly those representing changes in intestinal wall barrier and permeability, to determine whether these could be useful biomarkers of gut injury. This article reviews current and newly proposed markers of gut injury, the available literature evidence, recent advances and considers how effective they are in clinical practice. We discuss each biomarker in terms of its effectiveness in predicting NEC onset and diagnosis or predicting NEC severity and then those that will aid in surveillance and identifying those infants are greatest risk of developing NEC.

## Introduction

Necrotising Enterocolitis (NEC) remains one of the most significant complications of prematurity (1) with a high mortality and morbidity rate. It often presents acutely and causes rapid deterioration. For this reason, biomarkers of gut tissue injury have been examined for over 20 years with the hope of identifying non-invasive reliable markers that could predict the onset of NEC before clinical signs, allowing the opportunity for earlier interventions and improved clinical outcomes.

However, many of these biomarkers are non-specific or difficult and invasive to measure. In addition the main gastrointestinal diseases affecting preterm neonates are NEC and septic ileus; both trigger translocation of bacteria and luminal contents into the host related to intestinal permeability and impaired intestinal barrier function and it is often difficult to differentiate between them (2). Delays in diagnosis can result in surgery being performed when most of the gut is necrotic, resulting in short-bowel syndrome or withdrawal of care due to NEC totalis.

**Figure 1:**
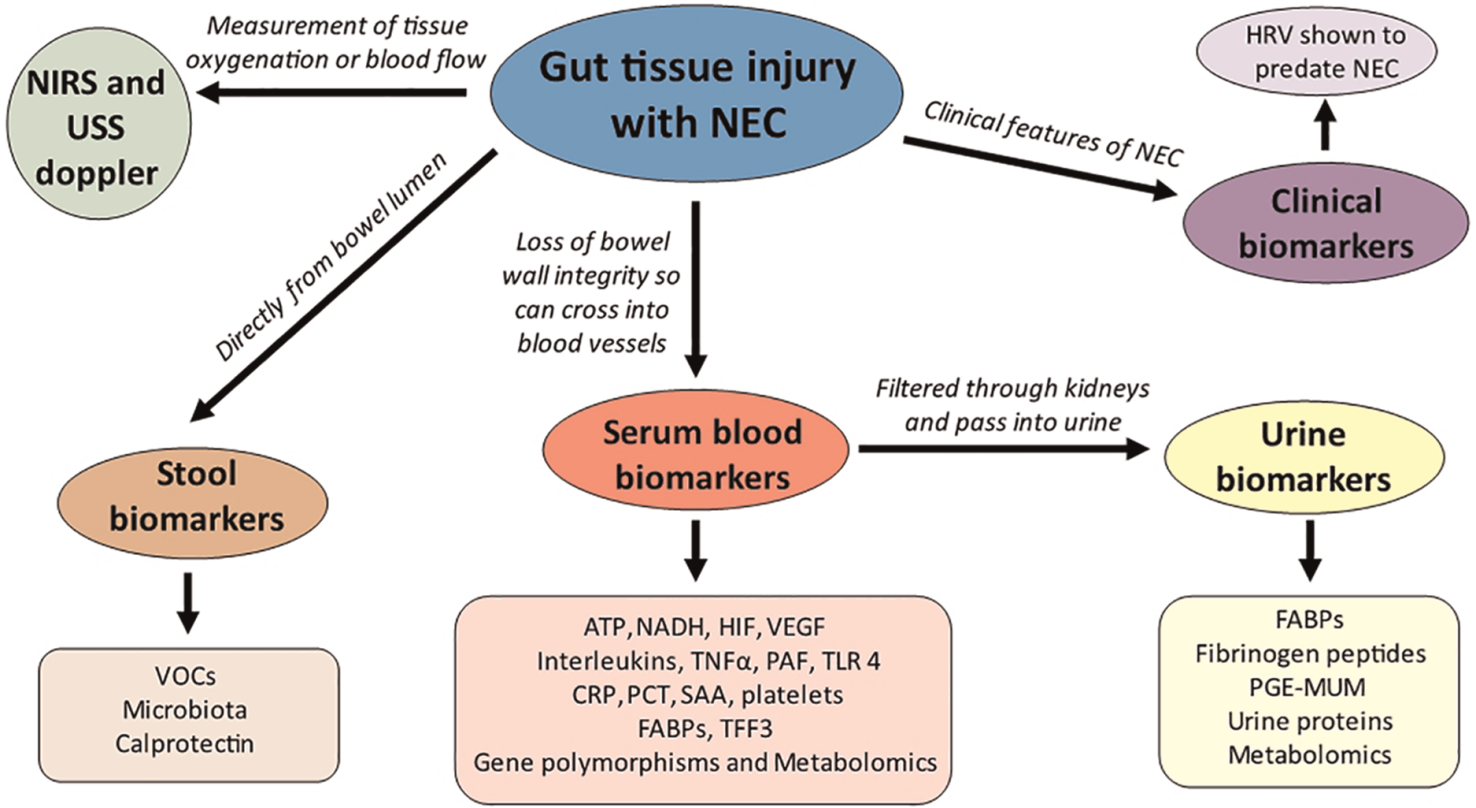
Non-invasive gut biomarkers and their origins (sample) of detection. ATP- adenosine triphosphate; CRP- C reactive protein; FABPs- fatty acid binding proteins; HIF-hypoxia inducible factor; NADH- nicotinamide adenine dinucleotide; NIRS- near infrared spectroscopy; PAF- platelet activating factor; PCT- procalcitonin; PGE-MUM- prostaglandin E2 Major urinary metabolite; SAA-serum amyloid A; TFF3- trefoil factor 3;TLR-4 toll like receptor 4; TNF*α*- tumour necrosis factor alpha; VEGF-vascular endothelial growth factor; VOCs-volatile organic compounds.

From a clinical perspective there are numerous approaches to finding a gut biomarker as a non-invasive measurement of gut injury rather than directly sampling the intestine, including stool, urine, and serum blood samples, as well as using clinical features known to predate NEC onset and newer emerging modalities such as Near Infrared Spectroscopy (NIRS) which reflects regional oxygenation (3) (Figure 1). Biomarkers range from non-specific biomarkers that form part of the proinflammatory and anti-inflammatory pathways of the immune system including C reactive protein and cytokines, non-specific gut biomarkers that can also be raised in other causes of inflammation such as calprotectin and volatile organic compounds and lastly more specific biomarkers that are associated with gut pathology which include fatty acid binding proteins (FABP) and trefoil factor-3 (TFF-3).

The ideal biomarker should have a modest sensitivity and high specificity in diagnosing NEC, would rapidly increase in response to disease onset, preferably prior to the onset of clinical signs, and decrease quickly in response to treatment and clinical improvement. In addition, the results should be available quickly to facilitate rapid decision making. Clearly for extreme preterm infants (those most at risk of NEC) minimising invasive and painful procedures, (e.g., venepuncture) is important, therefore another important criterion is to be non-invasive. Biomarkers of NEC are currently a hot topic in Neonatology as researchers endeavour to find ways which enable clinicians to detect this devastating condition earlier. There have been many reviews on this topic (4–10) which only serves to highlight just how important this topic is to the neonatal community. This narrative review focuses on the proposed tissue biomarkers but provides updates on more recently suggested biomarkers along with their benefits and pitfalls. We will examine recent advances, as well as newly proposed biomarkers, the “omics” era and clinical prediction models. We have aimed to discuss each biomarker in terms of the likelihood of it being used in clinical practice by examining their effectiveness in predicting NEC onset and diagnosis or predicting NEC severity or whether it will aid in surveillance and identifying those infants at greatest risk of developing NEC.

### Endogenous/exogenous biomarkers representing hypoxia injury

Direct measurement of tissue oxygenation and hypoxia *in vivo* is not possible therefore various surrogate markers have been proposed including some biomarkers in serum, such as serum lactate, which may represent the change of state of hypoxia in intestinal tissues (11–14). Tissue hypoxia can also be determined *in vivo* by looking at biochemical markers at the cellular level including decreased adenosine triphosphate (ATP) and increased nicotinamide adenine dinucleotide (NADH) (15). In hypoxia, hypoxia-inducible factor (HIF) up-regulates the transcription of various cytokines, including erythropoietin and vascular endothelial growth factor (VEGF) and while these have been proposed as markers of hypoxia and gut injury (16, 17) they are difficult to measure and not used in routine in practice. Table 1 summarises the tissue biomarkers of hypoxia and inflammation which have been proposed as predictors of gut ischaemia and injury (18–26), including their clinical use, advantages, and disadvantages, as well as the evidence for their use and any current clinical applications.

**Table 1:** Summary of the serological markers of ischaemia, hypoxia and inflammation which have been reported as potential biomarkers of gut inflammation/injury.

Biomarker	Theory	Literature evidence	Clinical use	Disadvantages	Advantages
Lactate	Tissue hypoxia causes metabolic acidosis due to anaerobic respiration to L-lactate D-lactate is a bacterial fermentation product that can translocate if gut permeability is increased	Grootjans et al. 2010 (3); Fredrickson et al. 2011 (4); Moller et al. 1996 (5); Lei et al. 2016 (6)	Used regularly in clinical practice to assess perfusion. Have been shown to be raised in preterm infants with NEC	Many reasons for lactate to be raised – low specificity	Easily and rapidly measured in practice D-lactate potentially more specific for bacterial overgrowth with gut permeability
ATP/NADH	Tissue hypoxia → decreased adenosine triphosphate (ATP) and increased nicotinamide adenine dinucleotide (NADH)	Carlet et al. 1996 (7)	Used in research	In vivo measurements at cellular level only	More accurate as measured directly at tissue level
HIF	Intrinsic marker for hypoxia	Vukovic et al. 2011 (8)	Used in research	Difficult to measure	
VEGF	Marker of tissue hypoxia	Tschirch et al. 2009 (9)	VEGF inhibitors used to treat diabetic macular oedema and Retinopathy of Prematurity	Protein transcription required for levels to increase therefore not useful in acute injuries and difficult to measure Invasive test	
ALT/AST/LDH	Serum markers of cell damage	Guzman-de la Garza et al. 2013 (10)	Measured routinely on bloods in neonates. Used in HIE to monitor liver injury	Non-specific Invasive test	Easy to measure
BNP	Cardiac natriuretic hormone animal studies suggest BNP protects intestinal tissues from endotoxin-induced hyper-inflammatory injury	Radwan et al. 2014 (11); Yang et al. 2014 (12)	Used in cardiology, not used for gut injury in neonates clinically, used in research	Difficult to measure Invasive test	
SMA	SMA is detectable in plasma after severe intestinal injury and is therefore a possible clinical marker of damage into deeper muscle layers	Evennett et al. 2014 (13)	Research only	Raised once significant deeper muscle was affected and therefore may not be useful in predicting early gut injury Invasive test Difficult to measure	
Cytokines	Part of the systemic inflammatory cascade. Inflammation underlies many causes of gut injury	Kliegman et al. 1981 (14); Guzman-de la Garza et al. 2013 (10)	Used in research rather than clinically	Non- specific and form part of the systemic inflammatory cascade. Invasive test	Relatively easy to measure
CRP	Marker of inflammation and infection	Pourcyrous et al. 2005 (15); Evennett et al. 2014 (13)	Widespread use for general marker of infection or response to treatment such as Antibiotics	Invasive test Non-specific marker of systemic inflammation	Easy to measure
PCT	Peptide precursor of calcitonin. Used as a marker of severe sepsis. Suggested as way to differentiate NEC from sepsis	Turner et al. 2007 (16)	Used in research currently NICE has recommended further research and data collection to show the impact of adding PCT testing to standard clinical practice	Not enough evidence to recommend that these tests are used in the NHS	Relatively easy to measure
SAA	Acute-phase protein produced by the liver and kidneys in response to inflammation	Reisinger et al. 2012 (17); Reisinger et al. 2014 (18)	Used in monitoring of some inflammatory diseases e.g., amyloidosis and other rheumatic diseases	Not enough evidence to currently use to predict NEC	Quick and easy to measure

ALT, (alanine aminotransaminase); AST, (aspartate aminotransferase); ATP, (adenosine triphosphate); BNP, (Brain natriuretic protein); CRP, (C reactive protein) LDH, (lactic dehydrogenase); NADH, (nicotinamide adenine dinucleotide); PCT, (procalcitonin) VEGF, (vascular endothelial growth factor).

### Inflammatory mediators as biomarkers of gut injury

#### Cytokines and cell surface receptors

Cytokines have an important modulatory role in intestinal inflammation and gut injury (22). Interleukins (IL) 1, 3, 6 and 8, tumour necrosis factor alpha (TNF*α*), platelet activating factor (PAF), expression of toll like receptor 4 (TLR 4) on cell surface have been linked with development of NEC. However, the inflammatory mediators are non-specific and form part of the systemic inflammatory cascade. Therefore their role as diagnostic tools in NEC is limited, particularly as the commonest differential diagnosis of NEC is septic ileus (18).

##### C-reactive protein (CRP)

The most used nonspecific biomarker is CRP, but the specificity is low, and it is not possible to differentiate NEC with sepsis, or other causes of inflammation, based on CRP alone. Pourcyrous et al. (23) evaluated the association between serum CRP and NEC. In their study fifty-five infants with stages II and III NEC had an abnormal CRP (out of 241 evaluated for GI symptoms), regardless of their blood culture results and this normalised within an average of 9 days unless complications developed. As suggested by the authors, this could advise treatment strategies in infants with suspected NEC in that if they have serial normal CRP values, antibiotics could be stopped, and feeds restarted. CRP becomes abnormal in stage 2 and 3 NEC meaning a raised CRP would be an indicator for treatment and a persistently raised CRP, despite adequate treatment, could suggest complications are developing. However its utility as a biomarker to diagnose NEC is limited because there could be several reasons for a raised CRP and Evennett et al. (27) found that although CRP is a sensitive marker for NEC, it is non–specific. Another limitation of using CRP as a biomarker for NEC is the lag of about 12–24 hours for a rise in CRP to occur after the onset of NEC. However, as it is a marker of active inflammation it does have a role in assessing the response to treatment and this is how CRP is used routinely in neonatal clinical practice currently.

##### Procalcitonin (PCT)

PCT is a peptide precursor of calcitonin, synthesised by the parafollicular C cells of the thyroid and involved in calcium homeostasis. PCT levels have been shown to rise within 3 hours in response to invasive infection (28). Turner et al. (24) reported low PCT levels during NEC in preterm infants; PCT levels were <1 ng/ml at presentation and <1.3 ng/ml thereafter in neonates with stage I and II NEC in comparison with 4.1 ng/ml in neonates with sepsis. It has been proposed as a potential biomarker to differentiate NEC from sepsis. Although there is not enough data for this to be implemented into routine practice, some centres are exploring its use in diagnosing sepsis through research studies.

##### Serum amyloid A (SAA)

Serum Amyloid A (SAA) is an acute-phase protein produced by the liver and kidneys in response to inflammation. Several studies have examined SAA levels and its association with NEC and suggested that it may be a potential marker for diagnosing severe or complicated NEC, especially if combined with other markers. Reisinger et al. (26) reported that levels of urinary SAA were significantly higher in complicated NEC; they demonstrated an optimised cut-off value of SAA of 40.7 ng/ml for the stage II and stage III NEC groups by Bells' modified criteria and a cut-off value between surgical and medical NEC was determined at 34.4 ng/ml with a sensitivity of 83% and specificity of 83%. This same group also reported that combining SAA levels with platelet count could improve sensitivity of identifying complicated or surgical NEC (94% sensitivity and 83% specificity). This suggests a potential role of SAA in diagnosing severe NEC, especially when combined with blood platelet counts. Given that mortality is higher with severe NEC or NEC requiring surgery, the ability to predict severe NEC to allow earlier and more aggressive therapeutic interventions would be important. However, SAA would need further validation before it could be used in clinical practice.

### Combinations of inflammatory markers

Cetinkaya et al. (29) measured SAA, PCT and CRP in diagnosis and follow up in 152 preterm infants with NEC. They demonstrated that PCT had the highest specificity (98%) and PPV (97%), but lowest sensitivity (92%) and NPV (94%) of the 3 markers. Elfarargy et al. (30) conducted a case control study of 20 healthy neonates and 20 with NEC and reported that there was an increase in faecal calprotectin and serum levels of CRP, PCT and epithelial neutrophil activating peptide-78 (ENA-78), in the NEC group in comparison to the control group. These studies add weight to the suggestion that we should be using a combination of multiple different biomarkers to help identify NEC earlier.

### More specific biochemical markers of gut tissue injury

The non-specificity of the above markers means they are unable to differentiate between sepsis, NEC, and other causes of gut inflammation. This has propelled researchers to focus on more specific gut tissue biomarkers. Table 2 summarises the gut tissue biomarkers proposed as possible predictors of NEC (25, 31–62) and we discuss their overall effectiveness below. Some of these are also affected by inflammation such as volatile organic compounds and calprotectin but are felt to be more specific than the inflammatory mediators above which is why we will discuss these biomarkers in this section.

**Table 2:** Summary of the main studies reporting on gut tissue biomarkers (FABPs, calprotectin, VOC and TFF) as predictors of necrotising enterocolitis (NEC).

**Gut biomarker**	**Author and year**	**Study design and population studied**	**Primary outcomes**
**FABPs**	Cheng et al. (2015) (23)	– meta-analysis of seven publications – 444 infants – literature search using Web of Science, Embase, Medline databases, Cochrane Library, CNKI, VIP, and other Chinese medical databases – QUADAS tool was used to assess the quality of the included studies	– pooled sensitivity of I-FABP was 0.67 for NEC Stage I, 0.74 for NEC Stage II, and 0.83 for NEC Stage III – pooled specificity was 0.84, respectively – AUC for each stage was 0.75, 0.82 and 0.91 respectively
	Coufal et al. (2016) (24)	– *n* = 42 – preterm infants – prospective study of preterm infants with suspected NEC – collected paired serum (at enrolment and week later) and urine (collected for two days in 6 hly intervals) samples	– levels of urinary I-FABP are higher in NEC patients than in sepsis patients or healthy infants with a sensitivity of 81% and a specificity of 100%
	Derikx et al. (2007) (25)	– *n* = 17 – confirmed NEC infants had mean GA 32 + 3 w and mean BW 1500 g – infants who subsequently did not have NEC had a mean GA of 30 + 4 w and mean BW 1487g – prospective study – over 18 months 17 consecutive infants were identified in whom NEC was suspected – urine was collected daily for at least one week	– five of the 17 infants with suspected NEC subsequently developed NEC stage IIa. Of the remaining 12 patients suspected of NEC, 7 had sepsis and the rest alternative gastrointestinal diagnoses – in the first urine sample, median urinary I-FABP:Cr ratio was significantly higher in neonates who ultimately developed NEC or intestinal necrosis (3.9 pg/nmol, range 2.3-6.6) than in those without NEC (1.2 pg/nmol, range 0.1–1.8) (*p* = 0.001) – no infant without either NEC or intestinal necrosis was found to have an I-FABP:Cr ratio >2 pg/nmol
	Edelson et al. (1999) (26)	– *n* = 31 infants with NEC – prospective pilot study – 3 plasma samples were obtained at symptom onset, after 8 and 24 h	– I-FABP was detectable in blood samples from all 7 infants with stage 3 NEC compared with 3 of 24 with stages 1 or 2 NEC – elevated plasma I-FABP concentrations were detectable before clinical staging could be made in 5 of the 7 subjects with stage 3 NEC
	Gollin et al. (2014) (27)	– *n* = 62 – preterm infants – GA 24–28 weeks – prospective study – urine collected daily – iFABPu was determined using an ELISA, and was expressed in terms of its ratio to urinary creatinine (Cr), i.e. iFABPu/Cr	– five infants developed NEC (3 had stage 2 and 2 had stage 3 NEC) and 21 age matched controls – the day before the first clinical manifestation of NEC iFABPu/Cru >10.2 pg/nmol predicted impending NEC with a sensitivity of 100% and a specificity of 95.6% – iFABPu/Cru did not predict NEC 2 days prior to the first sign of disease
	Guthmann et al. (2002) (28)	– *n* = 40 – preterm infants <33w CGA – prospective clinical study – control group (*n* = 26): infants between days 14 and 28 of age without abdominal symptoms or other clinical signs of illness – patient group (*n* = 14): infants who exhibited abdominal symptoms requiring a septic work up or an AXR – blood drawn at the initial evaluation to determine I- and L-FABP levels	– L-FABP concentration (median 7.6 ng/ml) was about 3 fold that of I-FABP (median 2.52 ng/ml) in plasma of healthy preterm infants – at onset of symptoms, I-FABP concentration was significantly higher in infants who later developed severe NEC compared to healthy infants and those, whose illness remained confined to stage I or II – L-FABP was significantly elevated compared to the control group at onset of symptoms regardless of the further course of NEC
	Heida et al. (2015) (29)	– *n* = 19 – infants with surgical NEC – combined data from prospective trials from two large paediatric surgical centres – 9 and 10 infants with surgical NEC were included from the two centres respectively – plasma and urinary I-FABP at disease onset were correlated with the length of intestinal resection during laparotomy	– length of bowel resection significantly correlated with I-FABP levels in plasma (Rho 0.68; *p* = 0.04 and Rho 0.66; *p* = 0.04) and in urine (Rho 0.92; *p* = 0.001)
	Li and Sheng (2017) (30)	– *n* = 135 – 3 groups with 45 infants in each group – prospective longitudinal study of I-FABP, TNF-α, and IL-6 – compared levels of each of them between three groups with 45 infants in each group: NEC patients, non-NEC patients, and healthy newborns – after matching day age, weight, gestational week and delivery mode serum TNF-α, IL-6 and I-FABP levels at 6, 24 and 72 h after admission were measured and compared with development of NEC	– the level of I-FABP in each time point in NEC was significantly higher than that in non-NEC group, and the level was the lowest in healthy group; the differences were statistically significant (*p* < 0.05)
	Schurink et al. (2014) (31)	– n = 37 – neonates with suspected NEC – 24 M, 13 F – median GA 28 w – median BW 1190 g – prospective observational study – plasma and urine I-FABP levels were measured at regular intervals during the first 24 h after onset of symptoms of NEC – assessed correlation between urine and plasma levels as well as with other parameters including IL-6 and lactate	– I-FABPu correlated strongly with I-FABPp (r0.80, *p* < 0.001) – very strong correlation between I-FABPu and I-FABPu/urine creatinine ratio (r 0.98, *p* < 0.001) – I-FABPu correlated significantly with serum IL-6 and lactate during the first eight hours of the disease
	Thuijls et al. (2010) (32)	– *n* = 226 – preterm infants before clinically suspected NEC – prospective cohort study – daily urinary I-FABP collected and infants monitored for signs of NEC developing	– 6 developed NEC – however, 4 out of these 6 did not have I-FABP levels above their proposed cut off for diagnosing NEC (2.2 pg/nmol creat) before clinical suspicion
	Yang et al. (2016) (33)	– 14 studies – 13 studies included in meta-analysis – 593 infants – literature search performed using PubMed and EMBASE – QUADAS-2 tool was used to assess the quality of the included studies – sensitivity, specificity and other measurements of accuracy of i-FABP were pooled	– sensitivities of urinary I-FABP and the I-FABP/Cr ratio for NEC were 64% and 78% and the specificities were 73% and 75%, respectively – sensitivity of serum I-FABP of 64% and 71% and a specificity of 91% and 76% for NEC and surgical NEC, respectively
**TFF**	Shi et al. (2014) (34)	– animal study of neonatal rats – *n* = 51 – 1 day-old Wistar rat pups -rats were killed on day 4, the distal ileum was harvested for morphological studies and the amounts of IL-1 *β*, IL-6, and IL-10 in the intestinal tissue were measured using ELISA – then TFF3 was given and cytokine levels remeasured	– TFF can decrease levels of inflammatory cytokines and enhance expression of anti- inflammatory cytokines
**Calprotectin**	Albanna et al. (2014) (35)	– *n* = 35 – preterm infants – 15 with NEC (mean GA 32 w, 9 M, 6 F) – 20 age/sex matched controls (10 M, 10 F) – case control study – collected stool samples from each group and compared the difference in level between the two groups	– significant increase in calprotectin in NEC group than controls (*p* < 0.001) – significant increase in faecal calprotectin with increasing severity of NEC (*p* = 0.016)
	Aydemir et al. (2012) (36)	– *n* = 50 – 25 with stage 2-3 NEC – 25 matched controls – prospective controlled study – stool samples were collected at the time of NEC diagnosis and 3–5 days later from the patients, and at similar postnatal age from controls	– median calprotectin levels were 1282 mcg/g for NEC infants at diagnosis compared to 365 mcg/g for controls – calprotectin significantly higher in NEC infants in first and second samples – calprotectin of 792 mcg/g was 76% sensitivity and 92% specificity for diagnosis of definitive NEC
	Bin-Nun et al. (2015) (37)	– *n* = 15 neonates ≤ 32w GA – 29 stool samples:13 from infants with NEC, 16 from infants with no NEC – stool samples collected for calprotectin analysis at 1 and 3 weeks postnatally and whenever there was clinical suspicion of NEC	– calculated a cut-off value (480 *μ*g/g) of fecal calprotectin that gave a maximum sum of diagnostic sensitivity and specificity – (sensitivity 100% and specificity 84.6%) – rapid assay calprotectin levels were elevated with NEC (3,000 µg/g stool [2075,7875] vs. without (195 µg/g stool [110,440] *p* < 0.001)
	Carrol et al. (2003) (38)	– *n* = 14 – divided into those infants with NEC (*n* = 7) and those without NEC as controls (*n* = 7) – both groups had a mean GA of 30 w – pilot study – identified 7 consecutive infants with clinical features of NEC treated with at least 7 days NBM, TPN and IV antibiotics – identified 7 controls matched for age, sex, and gestational age – stool samples obtained within 24 h of diagnosis of NEC	– infants with NEC had raised calprotectin at time of diagnosis compared with controls (288.4 mg/L and 98 mg/L respectively, *p* = 0.0006)
	Josefsson et al. (2007) (39)	– *n* = 59 – VLBW infants – 7 (disease group) developed severe abdominal disease defined as NEC or a condition leading to laparotomy – 52 controls – prospective cohort study – weekly calprotectin samples until week 8 of life	– in the control group median calprotectin levels were 332 mcg/g stool (range 12-9836) – in disease cases calprotectin was increased to >2000 *μ*g/g in 3 cases of NEC and 1 case of covered perforation with microscopic bowel inflammation – authors suggested >2000 mcg/g stool for cut off to diagnose NEC but 3 in NEC group didn’t reach that level (1 case of NEC without microscopic bowel inflammation and 2 cases of focal intestinal perforation)
	MacQueen et al. (2016) (40)	– *n* = 30 – infants with suspected NEC – prospective pilot study – eligible infants were those when an AXR was ordered to “rule-out NEC” – stool calprotectin was quantified at that time and in a follow-up stool – each episode was later categorised as NEC or not NEC – location of calprotectin in the bowel was determined by immunohistochemistry	– infants with NEC had higher initial and follow-up stool calprotectin levels than infants without NEC – calprotectin in bowel from neonates with NEC was within neutrophil extracellular traps (NETs)
** **	Nakayuenyongsuk et al. (2018) (41)	– *n* = 64 – preterm infants with a BW <1500 g without existing bowel disease – prospective observational cohort study – level IV neonatal intensive care unit from Oct 2015-Sept 2016 – stools were collected once daily for 30 days or until 32 weeks postmenstrual age	– 62% of infants had an initial stool sample with high baseline calprotectin levels (≥200 µg/g). – two patients in the cohort developed NEC – calprotectin levels for the entire cohort fluctuated during the observed period but generally increased in the third and fourth weeks after birth.
	Qu et al. (2020) (42)	– first meta-analysis of the diagnostic value of fetal calprotectin in NEC – *n* = 10 studies with 568 patients – searched several databases including PubMed, Medline, Web of Science and Cochrane Library – quality of included studies assessed by RevMan5 software (QUADAS-2)	– reported that the pooled sensitivity, specificity, diagnostic odds ratio (DOR) and AUC were: 0.86 (95% CI: 0.80–0.91), 0.79 (95% CI: 0.75–0.83), 34.78 (95% CI: 15.30 to 79.07) and 0.92 – performed subgroup analyses including those studies involving preterm infants and infants with stage II or higher NEC and they concluded that stool calprotectin was promising as a biomarker for NEC diagnosis, especially in preterm infants
	Reisinger et al (2012) (17)	– *n* = 62 – preterm infants – median GA 215 days – median BW 1328 g – prospective cohort study – in 62 consecutive neonates with clinical suspicion of NEC (29 with final diagnosis of NEC), urinary I-FABP, urinary serum amyloid A and fecal calprotectin were measured	– non-NEC median IFABP levels 1.2 pg/nmol, suggested 2.4 as cut off (sens 79%, spec 85%) – combining SAA with I-FABP did not increase diagnostic accuracy of I-FABP – combining calprotectin with I-FABP did increase diagnostic accuracy giving combined sens 94%, spec 79%, PLR 4.48 and NLR 0.08
	Rouge et al (2010) (43)	– *n* = 47 – preterm infants – median GA 29 w – median BW 1000 g – prospective cohort study – collected calprotectin stool samples at 2 weekly intervals until discharge	– 147 stool samples – median levels 138 mcg/g stool – wide range of inter- and intra-individual variation (coefficients of variation of 86% and 67%, respectively). – no statistically significant correlation between calprotectin and BW (*r* = 0.08, *p* = 0.59), nor GA (*r* = 0.09, *p* = 0.55) – significantly lower levels in those infants whose mothers had received antibiotics (*p* = 0.002) or if infant received postnatal antibiotics (*p* = 0.001) – those who were fed formula compared to breast milk had significantly higher levels (*p* = 0.02) – levels correlated positively with prominent gastrointestinal colonization by *Clostridium sp* (*p* = 0.019) and *Staphylococcus sp* (*p* = 0.047)
	Selimoglu et al. (2012) (44)	– *n* = 77 – cohort divided into groups of heathy term infants, healthy preterm infants, and those infants with NEC – prospective study – fecal lactoferrin and fecal calprotectin – levels were measured in 14 infants – with NEC and consecutively admitted – 40 healthy preterm, and 23 healthy – full-term newborns	– mean fecal lactoferrin and fecal calprotectin were not different between preterm and full-term infants (*p* = 0.235 and *p* = 0.845, respectively), or those who were diagnosed with NEC or not (*p* = 0.545 and *p* = 0.968, respectively) – stage of the disease did not have a statistical effect on mean levels (*p* = 0.694 and *p* = 0.267, respectively) – mean fecal lactoferrin and fecal calprotectin levels were not different in the breastfeed infants (*p* = 0.623 and *p* = 0.792, respectively).
	Van Zoonen et al. (2018) (45)	– *n* = 40 – infants with BW <1000 g or GA <30 w – 10 infants with NEC, 30 controls – prospective cohort study – measured stool calprotectin twice a week from day 1 of life until 5 weeks of age	– 163 stool samples – median calprotectin of whole sample was 332 micrograms/g stool (range <40 to 8230) – no difference in levels between NEC and controls – demonstrated wide intra-individual and inter-individual variation
	Yoon et al. (2014) (46)	– *n* = 16 – VLBW infants with GA <37 weeks – prospective cohort study – collected stools over two separate periods: Feb to April 2012 and May to June 2013 – stools collected from preterm infants with suspected NEC and normal preterm infants	– 154 stool samples – 4 infants developed NEC (12 stool samples) – significantly higher calprotectin levels in those in NEC group (*p* = 0.02) – no difference in the calprotectin levels according to the type and method of feeding between the NEC and non-NEC groups.
	Zhang et al. (2016) (47)	– *n* = 40 preterm infants – 17 NEC – 23 healthy controls – prospective observational study – measured calprotectin levels as well as clinical features, radiological findings, serological test, and other test results at diagnosis of NEC and compared with controls at same time	– median calprotectin levels in infants with NEC were significantly higher than those in the non-NEC group (858 (347.5, 1417.5) vs. 179 (125,265) μg/g, *p* < 0.001) – statistical difference in stage I, II, and III of NEC (*χ*2 = 6.672, *p* = 0.036), the median calprotectin levels were 457 (309, 875), 932 (532, 1712), and 3,108 (1378, 4276) μg/g, respectively – ROC curve defined a cutoff of 281 μg/g for NEC in preterm infants (*p* < 0.001), the AUC (95% CI) was 0.931 (0.804, 0.987), and the sensitivity and specificity were 88.2% (63.6%, 98.5%), 82.6% (61.2%, 95.0%), respectively
**VOC**	Berkhout et al. (2019) (48)	– *n* = 843 – preterm infants ≤30 weeks – prospective multicentre (9 neonatal intensive care units in the Netherlands and Belgium) trial – stool samples collected daily, up to the postnatal age of 28 days – VOCs measured by high-field asymmetric waveform ion mobility spectrometry (FAIMS) – VOC profiles of LOS infants (*n* = 127), up to 3 days prior to clinical LOS onset, were compared with profiles from matched controls (*n* = 127)	– VOC profiles show a significant difference up to 3 days before the onset of clinical symptoms of late-onset sepsis and allowed for preclinical discrimination between LOS and control infants – VOCs differed significantly between LOS cases and controls at all predefined time points – LOS caused by E. coli, S. aureus, and S. epidermidis could be differentiated from their matched controls with high predictive value, up to 3 days before the clinical onset
	de Meij et al. (2015) (49)	– *n* = 58 – preterm infants born at ≤30w GA – proof of principle prospective study across 3 neonatal intensive care units – stool samples collected daily, up to the 28th day of life – infants divided into 3 subgroups: NEC (*n* = 13), sepsis (*n* = 31), and matched controls (*n* = 14) – 3 time windows were defined: (1) T−5,−4 (5 and 4 days before diagnosis); (2) T−3,−2 (3 and 2 days before diagnosis); and (3) T−1,0 (day before and day of diagnosis of NEC/sepsis)	– stool VOC profiles of infants with NEC could significantly be discriminated from matched controls at T−3,−2 (AUC ± 95% CI, *p* value, sensitivity, specificity: 0.77 ± 0.21, *p* = 0.02, 83%, 75%); the accuracy increased at T−1,0 (0.99 ± 0.04, *p* ≤ 0.001, 89%, 89%) – VOC profiles of infants with NEC were significantly different from those with sepsis at T−3,−2 (0.80 ± 0.17, *p* = 0.004, 83%, 75%), but not at T−1,0 (0.64 ± 0.18, *P* = 0.216, 89%, 57%)
	El Manouni El Hassani et al. (2018) (50)	– *n* = 30 infants – prospective multicentre study – preterm infants <30 weeks’ gestation–admitted at one of nine participating neonatal intensive care units (NICU) in The Netherlands and Belgium – infants were born during the period of October 2014 and January 2018 – preterm born infants suffering from NEC, SIP, EOS and LOS were excluded – infants were divided into two subgroups based on enteral feeding composition postnatally: (1) breast milk (BM) fed (*n* = 15), which was defined as >75% of the total daily enteral feeding volume consisting of BM and (2) formula (FM) fed, which was defined as >75% of the total daily enteral feeding volume consisting of FM – stool samples examined from 7, 14 and 21 days postnatally – used eNose (Cyranose 320)	– although the numbers studied were small, they reported: – no differences in VOC patterns observed at the three time-points – combining the VOC profiles of these time-points resulted in a statistically significant difference between the two subgroups – in both subgroups, VOC patterns showed a stable longitudinal course within the first month of life
	Garner et al. (2009) (51)	– *n* = 13 – preterm infants – prospective pilot study – daily stool samples collected prospectively during an 8-month period – 6 infants subsequently developed NEC and were matched with 7 non-NEC infants – VOCs identified from the faecal samples taken before the onset of NEC and after diagnosis – faecal samples at similar ages were also studied from the control infants	– 224 different VOCs extracted from 65 samples – VOCs increased in number with age for non-NEC infants – in the days before and after the diagnosis of NEC there was a reduction in the number of VOCs – 4 specific esters present in controls—2-ethylhexyl acetic ester, decanoic acid ethyl ester, dodecanoic acid ethyl ester, and hexadecanoic acid ethyl ester—were consistently absent from all faecal samples in those infants who developed NEC
	Hosfield et al. (2020) (52)	– three groups were studied: (1) breastfed controls (*n* = 11), formula-fed controls (*n* = 15) NEC (*n* = 13) – five day old mice pups – experimental NEC was induced -breastfed and formula-fed control groups studied – after 4 days pups were euthanized – stool microbiome analysis by 16S rRNA sequencing – VOC analysis assessed by Cyranose 320 eNose device	– NEC pups had severe intestinal injury when compared to controls – microbiome analysis showed that both control groups had significantly higher microbial diversity and relative abundance of Lactobacillus than NEC, and lower relative abundance of Escherichia – VOC profile for NEC pups was significantly different from controls
	Mayor et al. (2014) (53)	– *n* = 1326 – preterm infants born at 23-24 w gestation – multi-centre study from eight UK neonatal – examined faecal samples taken using headspace GC–MS – built on work from pilot study by Garner et al above	– overall, when the entire cohort was analysed, a broad range of VOCs was found in 32 babies who developed NEC Stage – IIa or higher – 3 groups of VOCs including aldehydes, butanoic acid and methanedithiones strongly associated with increased risk of NEC
	Probert et al. (2019) (54)	– *n* = 1326 – preterm <34 weeks’ gestation – multicentre prospective study – 8 UK neonatal units – daily stool samples collected – 49 subsequently developed definite NEC – stool samples from 32 NEC cases were compared with samples from – frequency-matched controls without NEC – used gas chromatography–mass spectrometry for VOC measurement	– stool VOCs are altered in preterm infants with NEC – VOCs were found to cluster into nine groups with three being associated with NEC and indicated the possibility of disease up to 3–4 days before clinical diagnosis – discriminant analyses identified five individual VOCs, which are associated with NEC in babies at risk, each with an area under the receiver operating characteristics curve of 0.75–0.76, up to 4 days before clinical diagnosis – Certain VOCs were also found to play a protective role against development of NEC including 3-methyl and 2-methylbutanoic acid

AXR, (abdominal x-Ray); BW, (birthweight); CGA, (corrected gestational age); EOS, (Early Onset Sepsis); GA, (gestational age); I-FABP, (Intestinal fatty acid binding protein); IL-1 β, (interleukin 1 beta); IL-6, (interleukin 6); IL-10, (interleukin 10); L-FABP, (liver fatty acid binding protein); I-FABP:Cr, (Intestinal fatty acid binding protein to creatinine ratio); LIT, score (L-FABP, I-FABP and TFF-3); LOS, (Late Onset Sepsis); NEC, (Necrotising Enterocolitis); TFF-3, (trefoil factor 3); TNF-α, (Tumour necrosis factor alpha; VLBW, (very low birth weight); VOC, (volatile organic compounds).

#### Fatty acid binding proteins (FABPs)

Intestinal FABP (I-FABP) and liver FABP (L-FABP) are both found in intestinal mucosa; I-FABP is relatively specific for enterocytes whereas L-FABP is present in many tissues. Both are released when cell membrane integrity is damaged. They are tissue specific inflammatory markers elevated during ischaemia, acting as markers of enterocyte and/or hepatocyte death, that can be measured in serum or urine by enzyme-linked immunosorbent *assay (*ELISA) (63–65).

#### FABPs effectiveness at diagnosing NEC

FABPs have been one of the most widely studied biomarkers for NEC. Table 2 lists the main studies and several have demonstrated raised I-FABP concentrations in neonates with NEC (32, 34, 36, 38) correlating with onset and resolution of symptoms (66). FABPs are felt to be more promising for diagnosing NEC as they are only released with specific gut damage rather than with generic neonatal sepsis (67). In further support of its use as gut biomarker, I-FABP has been shown to correlate significantly with serum IL-6 and lactate during the first 8 h of the disease, which are associated with development of NEC (39). However, there are concerns that they would not be able to predict the onset of NEC prior to clinical symptoms. Thuijls et al. (40) study of 226 neonates before clinical suspicion of NEC supported the evidence that urinary I-FABP levels are not suitable as a screening tool for NEC *before* the onset of clinical symptoms raising the suspicion for NEC. Although Gollin et al. reported that elevated urinary I-FABP was a sensitive and specific predictive biomarker for NEC one day prior to the development of clinical manifestations, it could not predict NEC two days prior to the first clinical signs developing (35), meaning there is limited lead time in terms of warning of NEC onset.

#### FABPs effectiveness at predicting disease severity

However, they are promising in terms of levels predicting disease severity. Even with a small number of infants, several studies have reported significantly higher I-FABP values related to severity of NEC (33, 34, 36, 40, 66). Guthmann et al. (36) reported that at the onset of symptoms I-FABP concentration was significantly higher in infants who later developed severe NEC and Heida et al. (37) demonstrated that the length of bowel resection in infants with surgical NEC was correlated with I-FABP levels in both plasma (*p* = 0.04) and urine (*p* = 0.001), supporting the hypothesis that increased I-FABP levels correspond with the extent of necrotic tissue.

#### Using urinary or serum FABPs

Although serum I-FABP levels have been shown to be increased in infants with NEC, blood is never an ideal specimen for surveillance purposes, especially in preterm infants, because blood-taking procedures are painful and invasive. Plasma I-FABP concentrations correlate strongly with urine I-FABP levels meaning either urine or blood levels can be used but, in neonates, non-invasive urine specimens would be more preferable (39). Thuijls et al. (40) proposed that urinary I-FABP is a better biomarker than plasma I-FABP for diagnosing NEC because FABPs have a short plasma half-life and are readily excreted in urine. Total bowel necrosis could also prevent the release of I-FABP into the circulation giving a negative plasma test, whereas urinary I-FABP reflects the accumulation of I-FABP over time and has a higher chance of achieving positive urinary test results. Conversely, some infants with NEC are anuric in which case blood sampling might be necessary.

#### Potential pitfalls in using FABPs as a biomarker for NEC

A potential problem with I-FABP for diagnosis of gut injury in NEC is that, although NEC frequently affects the ileum and jejunum, it can be localised to the colon, or rarely, the stomach, in which case an increase in I-FABP might not occur, as expression is lower in these regions; > 90% of all I-FABP expression is in the ileum and jejunum (33). Furthermore, there are limited studies reporting normal ranges of FABP in neonates, especially in preterm infants and particularly in those born at 22–23 weeks gestation who are at the greatest risk of developing NEC. This therefore limits their use in predicting NEC as the cut off value which equates to NEC has not been conclusively identified, although some authors have suggested various cut off levels (25). Most available studies involve control infants without NEC and these infants' FABP levels can be reviewed to compare normal ranges. Shores et al. (68) examined 112 infants (24–40 weeks gestational age) who had specimens in the first week of life, but only 19 premature infants (24–29 weeks gestational age) who had longitudinal specimens. They reported that infants had low levels of I-FABP during the first week of life, independent of gestational age, and levels increased longitudinally in premature infants. However, this is a very small study to base any conclusions on and much larger studies are needed to establish both the normative data for infants and the cut-off levels for diagnosing NEC.

Some studies have measured the ratio between I-FABP levels in the urine (I-FABPu) and the urine creatinine to compensate for variation in urine concentration and some did not (25, 40, 69). However, few have determined whether it is necessary to do this; Schurink et al. (39) in their study of 37 neonates with a median gestational age of 28 weeks found a strong correlation between I-FABP measured in plasma and urine (r0.80, *p* < 0.001) and a very strong correlation between I-FABPu and I-FABPu/urine creatinine ratio (r 0.98, *p* < 0.001) suggesting that calculating urinary IFABP/creatinine ratio may not be needed. Another factor to consider when comparing published levels of I-FABP in neonates is that different ELISA kits have been used by different studies and, therefore, it is difficult to compare, especially as there is no standardised I-FABP preparations. In addition, I-FABP is currently only available as a research test and might not be suitable for rapid result availability.

#### Summary

A recent meta-analysis by Yang et al. (41) reported that I-FABP levels in plasma and urine have a high specificity (91% and 73% respectively), but moderate sensitivity (64% and 64% respectively), which limits their value as a NEC biomarker. Cheng et al. in 2015 (31) completed a meta-analysis on serum I-FABP and found that it had moderate accuracy for diagnosing NEC with a pooled sensitivity of 0.67 for NEC Stage I, 0.74 for NEC Stage II, and 0.83 for NEC Stage III. Both analyses showed that serum I-FABP has a high specificity for the diagnosis of NEC, but it is limited by its sensitivity, hindering it as a gut biomarker in preterm infants.

### Combining I-FABPs with other biomarkers

A recent review suggested that the diagnostic value of I-FABP can be improved by combining with other markers of intestinal injury (70) which adds weight to the theory that the best biomarker for NEC may well be a composite measure of many biomarkers in an algorithm. L-FABP, I-FABP and TFF3 are found in significantly higher levels in infants who develop confirmed NEC compared with those who do not (18, 40, 66, 67). Ng et al. (67) developed the LIT score which is the sum of combining the plasma level of each of these biomarkers – **L**-FABP, **I**-FABP and **T**FF3. Each biomarker is ranked 0–3 based on 3 cut offs to give a total LIT score of between 0 and 9. They reported significantly higher plasma levels of L-FABP, I-FABP and TFF3 and LIT score in patients with surgical NEC compared to nonsurgical cases and a significant association between these gut barrier biomarkers and mortality rate in the NEC group. With a median cut-off of 4.5 points, the LIT score identiﬁed surgical NEC with sensitivity and speciﬁcity of 83% and 100%, respectively.

FABPs have also been shown to correlate signiﬁcantly with non-speciﬁc biomarkers of inflammation including IL-6 (39) and IL-8 (71). Recently, combining with non-invasive markers including Near Infrared Spectroscopy (NIRS) has shown promise. Kalteren et al. (72) showed that blood transfusions in preterm infants are associated with concomitant signs of oxidative stress and intestinal injury as measured by raised levels of urinary 8-isoprostane and I-FABP respectively, in parallel with lower variability in splanchnic oxygenation and one could surmise that combing these markers could help predict NEC with greater reliability and certainty.

### Trefoil factors (TFF)

Trefoil factors 1–3 (TFF 1–3) are a family of polypeptides upregulated in response to gut mucosal injury which have a fundamental role in epithelial protection and repair. In NEC there is a down regulation of TFF3 expression leading to impaired mucosal regeneration (33, 36, 40, 66). Levels of TFF3 in plasma have been reported to be elevated in paediatric patients with sepsis (73), and in a hypoxia induced neonatal rat NEC model, TFF decreased inflammatory cytokines and enhanced expression of anti-inflammatory cytokines (42). This suggests those with lower levels of TFF would be at increased risk of developing NEC, but this is difficult and complex to measure and TFF have not been demonstrated to predict NEC onset in human studies.

### Stool volatile organic compounds (VOC)

Faecal VOCs, products of microbial metabolism in the gut, are proposed as a gut tissue biomarker of gut injury and NEC (56, 57, 59, 74) and VOCs have demonstrated potential as non-invasive early diagnostic biomarkers in other gastrointestinal diseases which have a common feature with NEC in that the intestinal microbiome is felt to play a part in the pathogenesis, including neonatal sepsis (56), inflammatory bowel disease (IBD) (75), and colorectal cancer (76).

Garner at al (59). reported that VOCs increase in number as the neonate matures, likely reflecting the growing diversity of intestinal flora. Faecal VOC profiles of infants with NEC significantly differ from controls and were reported to be present from 2 to 4 days predating the onset of clinical symptoms and therefore, have been postulated to be a potential biomarker of pre-clinical NEC (57, 59). However as de-Meij (57) highlighted, there is overlap in the VOCs between NEC and sepsis; infant profiles were not always distinguishable. Furthermore, studies are small as very few recruited neonates developed NEC (Table 2). El Manouni El Hassani et al. (58) conducted a prospective multicentre study of preterm infants <30 weeks' gestation and found no difference in VOC levels between infants with NEC and sepsis.

Probert et al. (62) recently conducted a multicentre prospective study looking at VOCs as potential markers for NEC in infants <34w gestation. They reported faecal VOCs were altered in preterm infants and could indicate the possibility of disease 3–4 days before onset of clinical symptoms. Although this was the largest prospective study to date it still is insufficient to recommend routine VOC measurement to predict NEC. One major limitation is that different techniques have been used when studying VOCs and there is no guideline for the exact bioanalytical method, although headspace gas chromatography-mass spectrometry is thought to be the gold standard for quantitative analysis of VOCs. El Manouni el Hassani et al. (77) recently proposed a protocol for VOC analysis which would potentially allow for a standardised analysis of VOCs which could only serve to improve their use in clinical practice.

However there remain many challenges to overcome before implementing routine point of care testing of VOC for clinical diagnoses. The patients included in the human studies of VOC (Table 2) were of different gestational and chronological ages, located at different hospitals, and were on different enteral feeds, all of which are known to affect VOC measurement (60). El Manouni El Hassani et al. (77) demonstrated that there is a significant difference in VOC profiles over the first 21 days of life in the 15 infants <30w gestational age that they studied which would limit the diagnostic use of the eNOSE, as this device does not identify VOCs present, but instead gives an overview of the total number and mixture of VOC present in a sample (78). El-Metwally (79) demonstrated other confounding factors including differential environmental exposures or therapeutic interventions, which are commonplace in preterm infants who have varying antibiotic courses and incubator humidity settings, which will vary with preterm infant gestation, can affect VOC levels (80). Course et al. (78) in their systematic review examine in detail other issues with using VOC such as the measurement repeatability in neonates. One further potential limitation to routine VOC analysis by headspace gas chromatography-mass spectrometry is the specialised equipment necessary.

### Stool calprotectin

Intestinal inflammation is characterised by the sequestration of neutrophils into the gut wall. Calprotectin is a neutrophil derived protein present in stool that is promising as a non-specific biomarker of gut injury because it is resistant to degradation and is stable in stool kept at room temperature (81). Table 2 reviews the studies that have examined the use of calprotectin in detecting NEC; the majority are of a small sample size and there is debate regarding its usefulness; some authors have reported raised calprotectin levels in infants whom have NEC (43, 44, 46, 48, 54, 55, 82), compared to well preterm infants, but others have reported no difference (52, 53).

Although calprotectin is in clinical use in some gastroenterological conditions, e.g., inflammatory bowel disease (83, 84), measuring calprotectin levels in NEC has no current practical clinical use, although a point-of-care test is available. Two recent studies have also questioned the utility of calprotectin in NEC, one of them using a point of care test (49, 53). In each of these, calprotectin was measured serially over time in preterm infants, and it was reported that calprotectin levels were much higher in preterm infants than in adults and that there are significant inter- and intra-individual variations in preterm infants during the first few weeks of life, limiting its utility in differentiating infants with NEC from those without. Furthermore, often neonates with NEC do not pass stool and this would limit the use of any stool biomarker in predicting NEC. More data is currently needed to determine whether a rise in calprotectin levels could predict NEC before clinical symptoms arise, and to decide on what cut off value should be used as various values have been proposed (Table 2). Interestingly, the previous cut off values for diagnosis of NEC that have been proposed range from 200 μg/g by Carroll et al. (46) to 2000 μg/g by Joseffson et al. (47). but in Van Zoonen's (53) study, all of these cut offs were within the range of concentrations observed in their control infants.

Pergialiotis et al. (85) conducted a systematic review of stool calprotectin levels as a non-invasive marker for NEC. This review included 13 studies with a total of 601 neonates and showed that the sensitivity and specificity of stool calprotectin as a diagnostic marker were 76%–100% and 39%–96.4%, respectively. Given it is a non-specific marker, combining it with other biomarkers is likely to increase its specificity, but as to what combination of biomarkers would be best is still unknown. Qu et al. (50) recently conducted a meta-analysis of the diagnostic value of fetal calprotectin in NEC looking at a total of 10 studies with 568 patients and reported that stool calprotectin was promising as a biomarker for NEC diagnosis, especially in preterm infants.

More longitudinal data in infants with NEC are necessary to determine whether acute rises in stool calprotectin levels before the onset of clinical symptoms can be confirmed as a diagnostic biomarker, either in isolation, or combined with other markers. However, it is unlikely that calprotectin could ever be used to *predict* NEC onset because it only rises in inflammation and once inflammation has occurred the NEC disease process has already started.

### New urinary biomarkers

In urine, with the aid of liquid chromatography-mass spectrometry (LCMS), other biomarkers have been identified and validated (fibrinogen peptides: FGA1826, FGA1883, and FGA2659) and Sylvester et al. (86) demonstrated that they could discriminate surgical NEC from nonsurgical NEC with these peptides and perhaps more importantly they also demonstrated in their study of 555 infants suspected of having NEC that the integration of clinical parameters *with* urine biomarkers in an ensemble model resulted in the correct prediction of NEC outcomes in all cases tested. However, LCMS is expensive and time consuming and as such currently has no place in routine clinical practice, but this evidence supports the idea that combining multiple biomarkers with clinical features into an algorithm may well be the future of predicting NEC.

Prostaglandin E2 Major Urinary Metabolite (PGE-MUM) has been described as a potential urine biomarker and is advantageous as it is stable in urine. Previous studies have demonstrated that it is raised in intestinal mucosal inflammation and reflects ulcerative colitis severity in paediatric patients (87). Konishi et al. recently examined its effectiveness as a surrogate marker for NEC and reported that PGE-MUM levels were higher in those with NEC, and correlated with improved status of NEC, length of necrotic intestine, and Bell's staging criteria. This is promising as both a diagnostic biomarker and a biomarker of severity, especially as urine markers would be preferred as they are non-invasive and more reliable than stool which is affected by the presence of an ileus for example. However, PG-MUM is non-specific as metabolites of cyclooxygenase are upregulated in all inflammatory conditions and levels could be affected by chronic lung disease (88). This was also the first study to report PGE-MUM levels in NEC and was of a small study size, therefore before any conclusions can be made larger studies are needed.

Various urine proteins have been examined as candidates for possible biomarkers of NEC. Recently a panel of 7 such urine proteins was identified by LCMS and subsequently validated by enzyme-linked immunosorbent assay in a study of 119 preterm infants (85 NEC, 17 sepsis, 17 control) (89). The 7 urine protein studied were alpha-2-macroglobulin-like protein 1 (A2ML1), cluster of differentiation protein 14 (CD14), cystatin 3 (CST3), pigment epithelium-derived factor (PEDF), retinol-binding protein 4 (RET4), and vasolin (VASN). Various combinations of these proteins were examined; A2ML1, CD14, CST3, PEDF, RET4, and VASN combined produced an area under the curve (AUC) of 98.4% for distinguishing medical and surgical NEC demonstrating their potential as markers of disease severity with medical NEC being less severe. A panel consisting of CST3, PEDF, and RET4 produced an AUC of 98.2% for distinguishing NEC from sepsis, which is the main differential for NEC given they present quite similarly in the initial stages in preterm infants. This study also adds more weight to the theory that combining several biomarkers is likely to improve their effectiveness at diagnosing NEC itself or predicting disease severity.

### Other markers focusing on intestinal wall barrier and permeability

Bischoff et al. explained the importance of the intestinal barrier (2) and recently there has been a move to look at the assessment of intestinal barrier function and permeability in humans by using intestinal permeability assays, and the assessment of biomarkers of epithelial integrity, including soluble adhesion molecules and Claudins. Claudins are tight junction proteins which are expressed in intestinal cells and therefore raised levels could imply intestinal epithelial cell damage. Only two studies have examined Claudins, and they involve a very small number of infants. Both studies found decreased claudin−2 expression in the intestinal tissue of infants with NEC (90) and a spike of increased excretion of claudin 3 in the urine (40). Thuijls et al. (40) examined urinary claudin-3 with urinary I-FABP and faecal calprotectin (FC) in 35 neonates suspected of having NEC and reported that median I-FABP, claudin-3 and FC levels were significantly higher in neonates with NEC than in neonates with other diagnosis. Blackwood et al. (90) examined urinary claudin-2 in six neonates and demonstrated raised urinary claudin-2 protein in those with NEC compared to those neonates without NEC (*p* < 0.001). However, these studies are too small to draw any conclusions regarding the effectiveness meaning there is currently not enough evidence to support Claudins as a biomarker of NEC as much larger studies are needed.

Citrulline is an amino acid not incorporated into proteins but produced by small intestinal enterocytes from glutamine and has been proposed as a marker of functional enterocyte mass. Loss of small bowel epithelial cell mass results in impaired intestinal permeability which is detected by reduced plasma citrulline levels. It has been demonstrated as a marker for chemotherapy-induced mucosal barrier injury in paediatric patients (91) but is not currently used routinely.

## New and upcoming biomarkers – the omics era

With increasing advances in technology there is a new era of using metabolomics and proteomics to look for new biomarkers for NEC (92–94) and gene polymorphisms are also being investigated with several being identified as being associated with NEC or NEC severity (95–97).

Agakidou et al. (98) have already reviewed the data on prediction, diagnosis and prognosis of NEC using a metabolomics and proteomics approach and this data is limited. Metabolomics detects the direct result of a biochemical response to a stimulus and metabolites are the final products of gene transcription. One benefit of such an approach is that numerous samples can be used including urine and plasma/serum, but also other bodily fluids. Numerous metabolomics have been examined in experimental models of NEC and these are suggested to be used as potential biomarkers for NEC. Agakidou et al. (98) reviewed the literature evidence for these in their article and although there is evidence of altered metabolomics in infants with NEC, these are mostly involving only small studies and much larger studies are needed before any conclusions can be accurately drawn. Furthermore, accurate identification of metabolites requires confirmation with known standards which also limits their current use in clinical practice.

Genomics is the study of all genes in an organism and there is some data suggesting that there is a genetic predisposition to NEC. This has led to the study of the preterm infant genome looking at specific genes encoding factors known to be involved in the pathogenesis of NEC including variants in the genes encoding nucleotide binding and oligomerization domain like receptors, autophagy-related protein, angiotensinogen, IL8, low-molecular-weight kininogen protein, (KNG1) Acetyl-CoA Carboxylase Beta, and CAT- Catalase, genes regulating the pathways of the receptors TLR2, TLR4, and TREM1 (96, 97, 99–101), as well as rs1048719 polymorphism in the intron region of the GM2 activator (GM2A) gene, the rs2075783 polymorphism in the exon 1 region (95) and SIGIRR variants (97). However currently the accuracy of genomic biomarkers in diagnosing NEC has not been examined; it is not known whether these associations would have any clinical use and there is the disadvantage of the time taken for the results of any genetic polymorphism tests which may preclude them from being a biomarker for diagnosing NEC early. However, they could be used to test infants at birth to help in predicting those who are at higher risk of NEC so that for example higher risk feeding strategies could be implemented. In addition, assays for genetic markers are expensive, time consuming and not currently available for clinical use. It is well known that the pathogenesis of NEC is multifactorial, and a lot of the proposed genetic markers above could also reflect secondary inflammatory responses, meaning they may not be specific markers as these inflammatory markers are common to other pathologies which may mimic NEC clinically including its main differential, septic ileus. It is not known to what extent environmental factors could modify the impact of these gene polymorphisms which will also hinder their effectiveness as biomarkers of NEC.

Transcriptomics is the study of the transcriptome, i.e., the study of the mRNA within a cell or organism. Experimental and clinical studies as well as a meta-analysis on long-non-coding RNA (lncRNA), micro RNA (miRNA), and mRNA profiles in NEC demonstrated a role of lncRNA, miRNA, and mRNA in NEC (102–106), but further evaluation in larger studies is needed. In a very recent study Pan et al. (107) completed a retrospective analysis on preterm pigs with NEC and found 344 differentially expressed genes between those piglets with NEC and control piglets and the authors suggested that blood gene expression analysis could be used to help identify early biomarkers of NEC, but clearly more research is needed and most importantly we need to see if this evidence in animal studies translates into human studies.

## Clinical prediction models

In addition, clinical factors have been explored such as heart rate variability (HRV) which reflects autonomic nervous system and tone; studies have shown that changes in HRV precede clinical symptoms of NEC by 2 days and the pattern of HRV change was also significantly associated with the clinical severity of NEC (stage II vs. stage III) (108). Doheny et al. conducted a prospective study in premature infants and found that low vagal tone (reflected by low HRV) in the first week of life in premature neonates may be a contributor for predicting the subsequent onset of NEC (109). However, HRV may be affected by many other conditions and so could not be used in isolation.

In 2015 Niemarkt et al. (9) reviewed the pathogenesis, diagnosis, and treatment of NEC and in this review discussed different clinical prediction models and biomarkers for NEC. Moss et al. (110) in their multicentre prospective, observational study examined clinical factors to see if any could predict severe NEC. Although they were able to identify 12 factors which were related to progressive NEC, they could not develop a clinical model to predict progression of NEC. Ji et al. (111) used a large multicentre prospective study to establish a clinical database and use machine learning taking into account clinical and laboratory results at the time of clinical presentation to create two NEC prediction models: the first to provide an automated diagnostic classification scheme and the second for risk-stratifying patients into low, intermediate and high NEC scores to determine the risk for disease progression and need of surgical intervention. If we could add biomarker data into such a model, then one would hope we could improve our diagnostic/predictive accuracy. Sylvester et al. (86) in their study looked at 27 clinical parameters and created a multivariate predictor of NEC progression. They reported that using clinical parameters alone resulted in a receiver-operator characteristic (ROC) curve with an area under the curve of 0.817 and left 40.1% of all patients in an “indeterminate” risk group. By adding in data from three urine peptide biomarkers (fibrinogen peptides: FGA1826, FGA1883 and FGA2659) they improved this ROC area under the curve to 0.856 and by combining clinical parameters with urine biomarkers their model correctly predicted NEC outcomes in all the cases the authors tested.

## Novel application of future biomarkers

Although there has been a plethora of studies examining biomarkers for detecting NEC the perfect biomarker has remained elusive and the current available data to support their use routinely is insufficient (10). It has however been demonstrated that the combination of clinical parameters with biomarker analysis may significantly improve our ability to identify individuals at risk of developing NEC and that combining markers themselves will improve diagnostic sensitivity and specificity. Identifying those individuals at higher risk early after birth for example with using the suggested dried blood analysis of gene polymorphisms will give time to allow for therapeutic interventions which could reduce the risk of NEC developing, therefore further work is needed in this area.

In addition, there are newer biomarkers being suggested which might more accurately reflect intestinal injury such as Near Infrared Spectroscopy (NIRS) (112–117) and USS doppler studies (118) of the splanchnic oxygenation and blood flow respectively. These alternative non-invasive modalities could facilitate earlier detection of the onset of NEC; however, they also have their own limitations. Future work needs to combine the existing most promising biomarkers including FABP and newly identified ones which have been shown to predict NEC in small studies, such as urine proteins. Only with combining multiple biomarkers from multiple modalities ranging from NIRS, urine, and stool biomarkers, as well as clinical parameters, we may be able to finally make progress in detecting NEC earlier to allow for prompt intervention. Future research needs to focus on these novel markers being combined with those that are more established and aim to develop a practical point of care test that could be used easily and quickly in clinical practice.

## Conclusion

Despite advances in neonatal care, the morbidity and mortality of NEC remain high; research must continue to look for ways to identify NEC sooner. Researchers have explored various tissue biomarkers to predict, diagnose and prognosticate NEC in newborn infants. Inflammatory mediators such as cytokines, CRP, PCT, SAA and combinations of these inflammatory markers have been investigated extensively but they are not useful in predicting or diagnosing NEC. However, CRP is used in clinical practice to measure the response to treatment of NEC.

Blood and urine FABPs, stool VOC and calprotectin are well known and studied gut tissue injury biomarkers. Of all the biomarkers evaluated for both predicting and diagnosing NEC the most promising are felt to be FABPs, however, their diagnostic accuracy is relatively low, most studies involve small numbers and are not randomised control trials. Researchers are investigating the usefulness of new biomarkers including urinary fibrinogen peptides and proteins, intestinal barrier function and permeability by using intestinal permeability assays, and the assessment of epithelial integrity including soluble adhesion molecules and Claudin. Further research on metabolomics and proteomics, and gene polymorphisms in predicting and diagnosing NEC is also needed to see if these could allow detection of those infants at the highest risk of developing NEC after birth. The ideal would be that these infants could then undergo greater surveillance for the possible onset of NEC and then subsequently combining this with the examination of multiple gut biomarkers aiming to detect the onset of NEC prior to its clinical manifestations.

Although no single biomarker has been identified, perhaps a combination of biomarkers would help. Further research is needed to see if we can develop a machine learning prediction and diagnostic model by inputting all the known biomarkers, combined with known clinical parameters suggestive of NEC to accurately predict NEC, which then improves outcomes. Using biomarkers in this way could facilitate the era of individualised medicine that we are approaching and mean that not only would be able to identify those at-risk neonates, but we could target them to receive maximal preventative therapies including utilising higher risk feeding regimens. Ultimately being able to identify NEC before clinical onset, would allow the opportunity for earlier intervention and the potential for improved clinical outcomes. Significantly improving the morbidity and mortality associated with NEC has so far proved elusive despite our best efforts, and the neonatal community must continue to strive to achieve this.
